# P-355. HIV Viral Blips and Low-Level Viremia Among People with HIV on 2-Drug versus 3-Drug Antiretroviral Regimen: A Matched Case-Control Study

**DOI:** 10.1093/ofid/ofaf695.573

**Published:** 2026-01-11

**Authors:** Hilal Abdessamad, Kristine Allen, Wassim Jamaleddine, Andres Bran Acevedo, Dima Dandachi

**Affiliations:** University of Missouri, Columbia, Missouri; University of Missouri, Columbia, Missouri; Central Michigan University, Columbia, Missouri; University of Missouri, Columbia, Missouri; University of Missouri - Columbia, Columbia, MO

## Abstract

**Background:**

As 2-drug antiretroviral therapy (ART), including both oral and long-acting injectables, are increasingly utilized, it is unclear whether differences exist in the prevalence of viral blips (VB), low-level viremia (LLV), and their clinical outcomes compared to 3-drug ART.

**Table 1:**
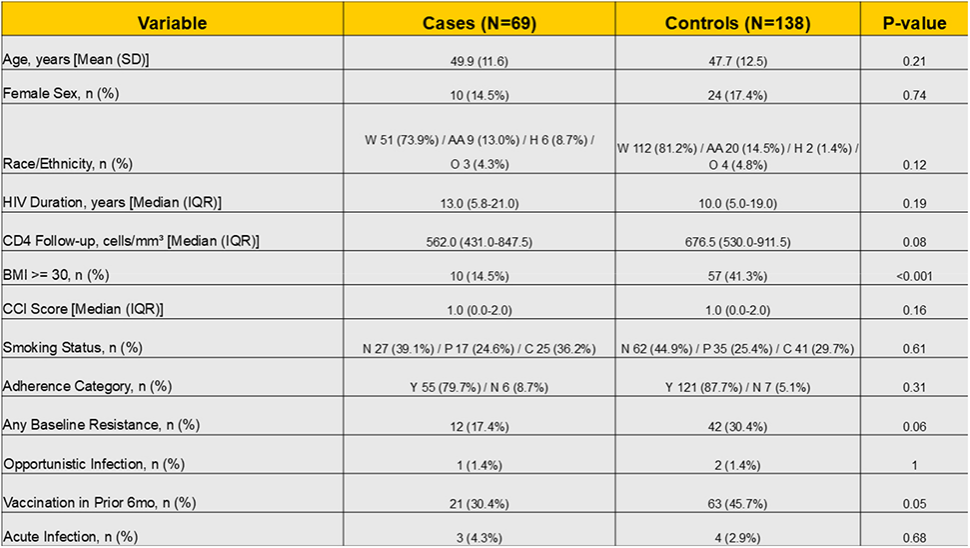
Baseline differences between cases and controls. Continuous variables presented as Mean (SD) or Median (IQR). Categorical/Binary as N (%). Race/Ethnicity order: White (W) / African American (AA) / Hispanic (H) / Other (O) Smoking Status order: Never (N) / Past (P) / Current (C). Adherence Category order: Yes, adherent (Y) – No, non-adherent (N) P-values are from unadjusted tests (Welch's t-test, Mann-Whitney U, Chi-square, or Fisher's Exact Test).

**Table 2:**
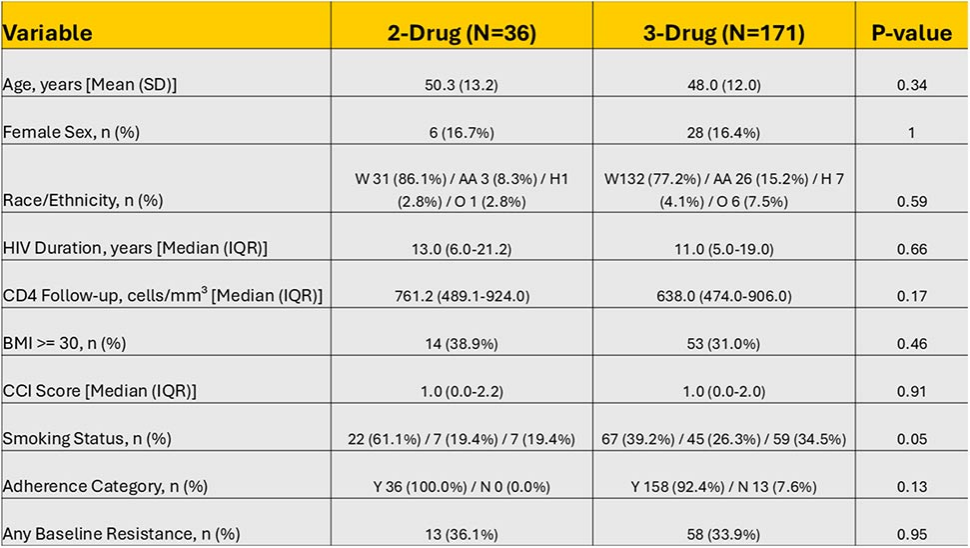
Bivariate analysis results for differences between PWH on 2-drug vs 3-drug ART. Continuous variables presented as Mean (SD) or Median (IQR). Categorical/Binary as N (%). Race/Ethnicity order: White (W) / African American (AA) / Hispanic (H) / Other (O) Smoking Status order: Never (N) / Past (P) / Current (C). Adherence Category order: Yes, adherent (Y) – No, non-adherent (N) P-values are from unadjusted tests (Welch's t-test, Mann-Whitney U, Chi-square, or Fisher's Exact Test).

**Methods:**

We performed a matched case-control study to compare adult People with HIV (PWH) on 2-drug vs. 3-drug ART seen between 2018 and 2025 in a single HIV clinic in Missouri. VB is 1 detectable VL < 400 copies/mL followed by suppression and LLV is ≥ 2 VL above detection and < 200 copies/mL after achieving virologic suppression on ART. Cases defined as having either a VB or LLV. Controls had undetectable VL. PWH with no 2 consecutive VL available were excluded. Mahalanobis distance matching was used on a 1:2 ratio, on baseline characteristics, CD4 count, and duration of HIV infection. A bivariate analysis was conducted to detect differences between PWH who were on 2-drug vs. 3-drug ART. Missing values were imputed. Conditional logistic regression was used for statistical analysis. The primary outcome was the presence of VB/LLV. Secondary outcomes, assessed among cases after a year of follow-up, included virologic failure (VF), continuous LLV or a suppressed VL on the same or different ART regimen.

**Figure 1:**
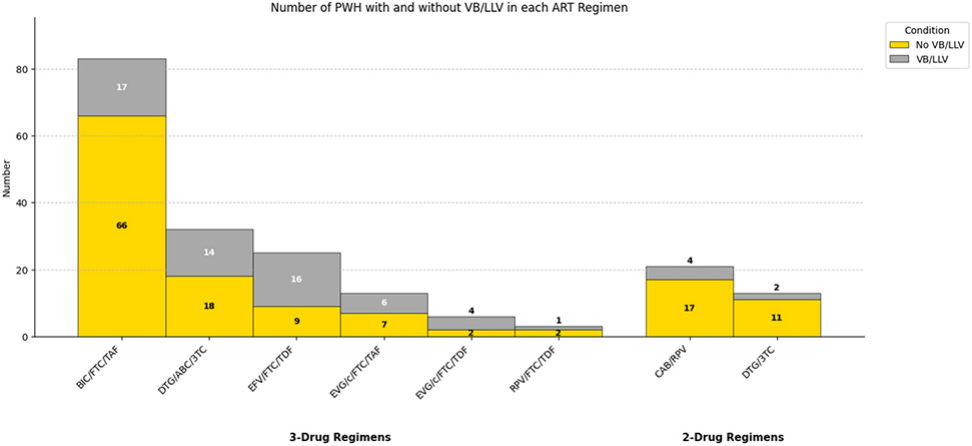
Number of PWH with and without VB/LLV in the most common ART Regimens. BIC/FTC/TAF (Bictegravir/Emtricitabine/Tenofovir Alafenamide), DTG/ABC/3TC (Dolutegravir/Abacavir/Lamivudine), EFV/FTC/TDF (Efavirenz/Emtricitabine/Tenofovir Disoproxil Fumarate), EVG/c/FTC/TAF (Elvitegravir/Cobicistat/Emtricitabine/Tenofovir Alafenamide), EVG/c/FTC/TDF (Elvitegravir/Cobicistat/Emtricitabine/Tenofovir Disoproxil Fumarate), RPV/FTC/TDF (Rilpivirine/Emtricitabine/Tenofovir Disoproxil Fumarate), CAB/RPV (Cabotegravir/Rilpivirine), and DTG/3TC (Dolutegravir/Lamivudine).

**Results:**

We included 207 PWH, 69 cases and 138 controls. Except for BMI, there were no significant baseline differences between cases and controls, including adherence and resistance genotypes (Table 1), and there were no significant differences between PWH on 2 vs 3 drug ART (Table 2). Among the 69 cases with VB/LLV, 7 (10.1%) were on 2-drug ART, compared to 29/138 (21%) in controls (Figure 1). Our analysis indicated a trend towards lower odds of VB/LLV on PWH on 2-drug ART, not statistically significant. Matched Odds Ratio: 0.43 (95% CI 0.18 – 1.03, P=0.059). Of the 69 cases 62(90%) were re-suppressed on the same ART, 4 (5.7%) were re-suppressed on a different ART, 3 (4.28%) had continuous LLV and none had VF.

**Conclusion:**

Our data suggests no statistically significant difference in the odds of VB/LLV and the rates of virologic suppression between 2-drug ART as compared to 3-drug ART. Given the limited data on this topic, larger multi-centered studies are needed for a more powerful assessment of this relationship.

**Disclosures:**

Dima Dandachi, MD, MPH, Gilead Sciences: Grant/Research Support|Gilead Sciences: Honoraria|ViiV Healthcare: Grant/Research Support|ViiV Healthcare: Honoraria

